# Anthropometric indices of First Nations children and youth on first entry to Manitoba/Saskatchewan residential schools—1919 to 1953

**DOI:** 10.3402/ijch.v75.30734

**Published:** 2016-06-27

**Authors:** F.J. Paul Hackett, Sylvia Abonyi, Roland F. Dyck

**Affiliations:** 1 Department of Geography and Planning, University of Saskatchewan, Saskatoon, SK, Canada; 2 Department of Community Health and Epidemiology, University of Saskatchewan, Saskatoon, SK, Canada; 3 Department of Community Health and Epidemiology, University of Saskatchewan, Saskatoon, SK, Canada; 4 Department of Medicine, Canadian Center for Health and Safety in Agriculture, University of Saskatchewan, Saskatoon, SK, Canada

**Keywords:** First Nations, indigenous, BMI, children, youth, residential school, diabetes, obesity, underweight, weight for age, height for age

## Abstract

**Background:**

First Nations people are experiencing increasing rates of obesity and type 2 diabetes but no anthropometric information exists from before the 1950s to provide context to these epidemics.

**Objective:**

To compare anthropometric indices of First Nations children and youth on first entering residential schools with historical and contemporary reference groups.

**Methods:**

This observational cross-sectional study used archival records from the Department of Indian Affairs to calculate body mass index (BMI), height for age (HA) and weight for age (WA) of all known children and youth undergoing physical examinations on first entering residential schools in Saskatchewan and Manitoba from 1919 to 1953. Proportions of children and youth in each BMI category were determined by age, sex, time period and residential school. Z-scores for HA and WA were determined by age group and sex. Finally, median heights and weights were compared with a non-Indigenous cohort from the 1953 Canadian survey.

**Results:**

On admission to residential schools, 1,767 First Nations children and youth (847 boys, 920 girls) were more likely to have normal BMIs (79.8%) than Canadian children and youth today (66.5%), but lower rates of overweight/obesity (10.9% vs. 32.0%) and higher rates of underweight (9.3% vs. <2.0%). There was an overall trend of diminishing levels of underweight and increasing levels of overweight/obesity over time. Although 6.6% of boys and 7.9% of girls had HA Z-scores >−2, age-specific median heights tended to be higher than Canadian children and youth in 1953. Under 3% of children and youth had WA Z-scores of >−2.

**Conclusions:**

A large majority of First Nations children and youth exhibited normal anthropometric indices on first entering residential schools in Manitoba and Saskatchewan from 1919 to 1953. These historical findings provide an important context to the current epidemics of obesity and type 2 diabetes and suggest that the nutritional conditions in these First Nations children's communities were satisfactory during the residential school era.

Since the 1970s, Canadian children and youth have experienced substantial increases in the prevalence of overweight and obesity. Using body mass index (BMI) categories, the 2009–2011 Canadian Health Measures Survey reported that 32% of boys and girls aged 5–17 were overweight and obese, increasing from 15% among 2- to 17-year-olds in 1978–1979 (1,2). Most regional data for Indigenous children and youth indicate that current overweight and obesity rates are even higher in this demographic (3–8). Indeed, a recent meta-analysis of Canadian Aboriginal populations found that 29.8% of those <18 years were overweight and 26.5% were obese (9). This marked population disparity in the prevalence of overweight/obesity is an important risk factor in the epidemic of type 2 diabetes in both First Nations children (10–12) and young adults (13,14).

In Canada, 3 groups of Indigenous people are constitutionally recognized under the descriptor Aboriginal: First Nations, Inuit and Métis. Although early anecdotal reports and images suggest that elevated overweight/obesity prevalence is a recent occurrence among these populations, little anthropometric information exists for any groups of adults or children in Canada prior to the 1960s (15–17). Notwithstanding this paucity of data, we were recently able to access archival records of First Nations children and youth from 1919 to 1953 when they first left their home communities to attend residential schools in Saskatchewan and Manitoba. Under the residential school system, children were intentionally placed at a distance from their home communities in order to facilitate assimilation into the broader Canadian society (18). The system attempted to achieve this objective by disrupting the parenting process and limiting opportunities for children to be exposed to their Indigenous language and culture. Corporal punishment and other forms of abuse were widespread. Although attendance at the schools was voluntary during the early years, in 1920, the government amended the Indian Act requiring all First Nations children aged 7–15 to attend residential schools (18). Physical examinations of children, including height and weight measurement, were conducted shortly after the first entry to these institutions.

To provide historical context to the recent epidemics of obesity and type 2 diabetes among First Nations people, the primary objective of this study was to determine BMIs and BMI categories of First Nations children and youth from the pre-1950s and relate these findings to those of their contemporary Indigenous and non-Indigenous counterparts. Our secondary objective was to evaluate the overall nutritional status of these residential school children based on BMI as well as weight for age (WA) and height for age (HA).

## Methods

### Data source

This observational cross-sectional study examined BMI, HA and WA of children and youth on first entering residential schools in Saskatchewan and Manitoba from 1919 to 1953. Data were derived from microfilm copies of residential school entrance examinations filed with the Department of Indian Affairs in Ottawa (19). They included a general admission form recorded by school officials and a physical examination form completed by a physician. The physical examination was primarily intended to identify children with active tuberculosis and prevent the admission of such children to residential schools (20,21). The physical examination form included fields for age, sex, height and weight. However, no descriptions of weight and height measurements were provided, and it is not known whether the methods used were similar or consistent among schools and over time.

Because these data are publicly available, the University of Saskatchewan Research Ethics Board determined that a review was unnecessary. However, the manner by which this information was collected raises ethical sensitivities about its use that merit further consideration (see discussion). Most children and youth in this study were admitted to 1 of 5 Indian Residential Schools: Brandon Residential School (Methodist) in Brandon, Manitoba; Beauval Residential School (Roman Catholic), Beauval, Saskatchewan; Round Lake Residential School (Methodist), Whitewood, Saskatchewan; St. Anthony's Residential School (Roman Catholic), Onion Lake, Saskatchewan; and St. Barnabas Residential School (Anglican), Onion Lake, Saskatchewan. Smaller numbers of boys and girls entered Saskatchewan Indian Residential Schools in Delmas, Okanese and Prince Albert. These children and youth likely represent most of the total number entering those schools over the study period because, by 1911, federal government policy directed that all students undergo a physical examination prior to being admitted (18,22). However, since approximately 150,000 First Nation, Inuit and Métis children attended about 130 residential schools across Canada during the residential school era (23), the children and youth in this study represent only a small fraction of the total.

### Calculation and evaluation of anthropometric indices

Information from entrance physical examinations was transcribed and entered into a Microsoft Access database before transferring it to Microsoft Excel for final analysis. All information was de-identified and analysed in aggregate for the study. Height and weight were converted into metres and kilograms, respectively, and BMI was calculated using the formula BMI=(weight in kg)/(height in m^2^). Children and youth were placed into 5 BMI categories based on age-specific International Obesity Task Force cut-points corresponding to definitions of very underweight, underweight, normal weight, overweight and the obese among adults (24,25). Because the birth dates of the children and youth were not recorded, we used the mid-year BMI-for-year percentiles as the reference for each age recorded (for example, 7.5 for a child aged 7). Children and youth with no age, height and/or weight recorded were excluded from the analysis, as were individuals with implausible BMIs. The date of height and weight measurements was the date of the physical examination or, if that was illegible or not recorded, the date of school admission.

The proportions of children and youth in each BMI category were determined for the total population and by sex, age, age group, time period and residential school. We chose 1919–1929 (pre-depression), 1930–1938 (Great Depression), 1939–1945 (Second World War) and 1946–1953 (post-war) as the time periods since they corresponded to important national and international events. We also compared the median age-specific BMIs of residential school children and youth with the 50th percentile values of current World Health Organization (WHO) Growth Charts for Canada (26).

To assess the general nutritional status of residential school children, we used WA (27) and HA (28) Z-score calculators based on 1977 National Center for Health Statistics growth charts to determine the proportions of children by age and sex who were in >−2, −1 to −2, −1 to +1, +1 to +2 and >+2 Z-score categories. We further compared median age-specific heights and weights of residential school children and youth with data from the 1953 Canadian weight-height survey of predominantly non-First Nations children (17), as well as 50th percentile values from WHO growth charts. The 1953 survey data were used as a comparison because it is the earliest published record of weights and heights of Canadian children and closest in time to the residential school era. Because the 1953 survey did not provide individual data, we could not calculate BMIs, WAs or HAs from its results.

### Statistical analysis

Our analysis was primarily descriptive in nature because of its small subgroups and the lack of a suitable reference and control group, in most instances. Moreover, our study population is likely a complete population rather than a sample. For children and youth first entering residential schools, proportions in each BMI category were categorized by sex, age group, time period and residential school. We presented these data using a log scale to more clearly define BMI differences within and between groups. Comparisons of median height and weight between residential school children and youth and the 1953 Canadian height–weight survey were also descriptive in nature. However, we compared overall sex differences in BMI, WA and HA categories using χ^2^ tests and a significance level of p≤0.05.

## Results

There were 2,079 residential school admission forms from which information was extracted for the initial database. After removing duplicates and files with insufficient data, there were 1,767 children and youth (920 girls and 847 boys), who were first admitted to a residential school from 1919 to 1953, and whose information was available for further analysis. On admission, most children and youth were aged 7–10 (Table I), but admission age ranged from 4 years (18 individuals) to 19 years (1 individual). The schools with the largest number of admissions were Beauval (394), Brandon (285), Onion Lake (680) and Whitewood (187). However, we also included children and youth admitted to Delmas (55), Okanese (48) and Prince Albert (118).

**Table I:** Numbers (%) of residential school children/youth by age, time period, school site and sex

	Boys	Girls	Total
Age			
4	11	7	18
5	12	18	30
6	47	70	117
7	226	250	476
8	163	139	302
9	89	91	180
10	93	82	175
11	59	60	119
12	49	53	102
13	43	43	86
14	34	54	88
15	12	35	47
16	8	13	21
17	1	4	5
18	0	0	0
19	0	1	1
Total	847 (47.9)	920 (52.1)	1767
Age group			
4–7	296	345	641 (36.3)
8–11	404	372	776 (43.9)
12+	147	203	350 (19.8)
Time period			
1919–1930	76	81	157 (8.9)
1931–1938	320	271	591 (33.4)
1939–1945	199	217	416 (23.5)
1946–1952	251	351	602 (34.1)
School site			
Beauval	194	200	394 (22.3)
Brandon	115	170	285 (16.1)
Onion Lake	350	330	680 (38.5)
Whitewood	103	84	187 (10.6)
Delmas	27	28	55 (3.1)
Okanese	22	26	48 (2.7)
Prince Albert	36	82	118 (6.7)

The admission forms showed that 70–80% of children and youth in this study entered schools directly from their home communities, either with no previous schooling or having attended a local day school. Of the remaining 20–30%, some older students in particular were likely transferred from other residential schools. However, as most physical examinations were performed on a child's first enrolment to a particular school, anthropometric indices largely reflect conditions in the child's home community rather than conditions within the residential school system.

Table II shows the proportions of all children and youth in each BMI, WA and HA category by sex and age group. Overall, 82.0% of boys and 77.7% of girls had normal BMIs (p=0.024). The sex difference was largely due to a greater proportion of girls in the underweight category (6.9% vs. 4.3% of boys; p=0.017). Only 11.0% of girls and 10.8% of boys were overweight or obese. Although the numbers are small, they show a trend of increasing prevalence of underweight and decreasing prevalence of overweight/obesity with increasing age among both boys and girls.

**Table II:** Residential school children: BMI's, weight for age and height for age by age group and sex

	Age 4–7				Age 8–11				Age 12–16				All				Significance
					
	Boys		Girls		Boys		Girls		Boys		Girls		Boys		Girls		Boys vs. Girls
									
	296		345		404		372		146		198		846		915		
									
Number	n	%	n	%	n	%	n	%	n	%	n	%	n	%	n	%	*p*
**BMI**																	
Very underweight	5	1.7	9	2.6	13	3.2	21	5.6	7	4.8	10	5.1	25	3.0	40	4.4	0.115
Underweight	6	2.0	21	6.1	20	5.0	19	5.1	10	6.8	23	11.6	36	4.3	63	6.9	0.0167
Normal	219	74.0	253	73.3	348	86.1	306	82.3	127	87.0	152	76.8	694	82.0	711	77.7	0.0239
Overweight	60	20.3	46	13.3	19	4.7	23	6.2	2	1.4	11	5.6	81	9.6	80	8.7	0.545
Obese	6	2.0	16	4.6	4	1.0	3	0.8	0	0.0	2	1.0	10	1.2	21	2.3	0.076
**Weight for age**																	
>−2 Z-score	3	1.0	3	0.9	7	1.7	12	3.2	8	5.5	10	5.1	18	2.1	25	2.7	0.411
−1 to −2 Z-score	25	8.4	21	6.1	35	8.7	47	12.6	22	15.1	26	13.1	82	9.7	94	10.3	0.685
+1 to −1 Z-score	221	74.7	277	80.3	315	78.0	294	79.0	116	79.5	148	74.7	652	77.1	719	78.6	0.446
+1 to +2 Z-score	43	14.5	41	11.9	45	11.1	19	5.1	0	0.0	14	7.1	88	10.4	74	8.1	0.093
>+2 Z-score	4	1.4	3	0.9	2	0.5	0	0.0	0	0.0	0	0.0	6	0.7	3	0.3	0.262
**Height for age**																	
>−2 Z-score	29	9.8	34	9.9	17	4.2	22	5.9	10	6.8	16	8.1	56	6.6	72	7.9	0.313
−1 to −2 Z-score	44	14.9	51	14.8	70	17.3	60	16.1	32	21.9	28	14.1	146	17.3	139	15.2	0.239
+1 to −1 Z-score	174	58.8	215	62.3	259	64.1	236	63.4	95	65.1	135	68.2	528	62.4	586	64.0	0.478
+1 to +2 Z-score	35	11.8	29	8.4	42	10.4	39	10.5	6	4.1	18	9.1	83	9.8	86	9.4	0.769
>+2 Z-score	14	4.7	16	4.6	16	4.0	15	4.0	3	2.1	1	0.5	33	3.9	32	3.5	0.654

Table II also shows Z-scores for WA and HA by age group and sex. Overall, almost 80% of both boys and girls had WA Z-scores from −1 to +1. Only 2.1% of boys and 2.7% of girls had WA Z-scores >−2. As was noted for BMI, there was a trend for increasing proportions of children with negative Z-scores and decreasing proportions of children with positive Z-scores with increasing age.

Overall, 62.4% of boys and 64.0% of girls had HA Z-scores from −1 to +1. Compared to WA findings, a relatively larger proportion of both sexes had negative HA Z-scores with 6.6% of boys and 7.9% of girls in the >−2 category.

**Fig. 1:**
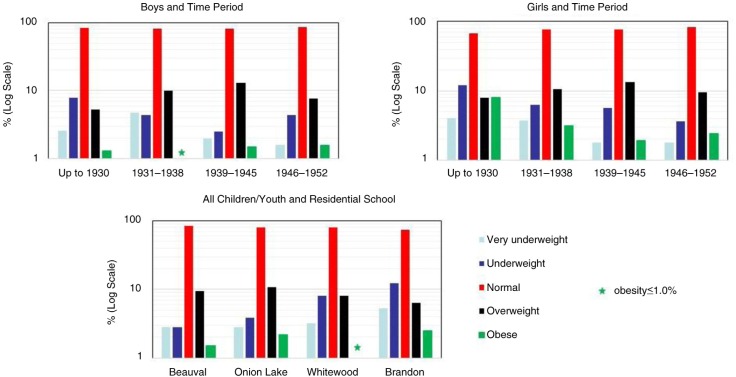
Body mass index categories of children first entering residential schools by time period and school site.

Figure 1 shows BMI categories by time period and school site. For both boys and girls, there was a trend for decreasing proportions of those in underweight categories and increasing proportions of those in overweight categories from pre-1931 to the war years (1939–1945). After 1945, there was a slight rebound in the underweight BMI categories for boys, and a small drop in the overweight categories for both sexes, which coincided with the highest proportion of children with normal BMIs. Over 82% of total children and youth and almost 85% of 6- to 8-year-olds fell into that category (the latter data not shown). Children and youth admitted to residential schools in the southern prairies (Whitewood and Brandon) were more likely to be in the underweight categories than those in west-central (Onion Lake) and north-western (Beauval) Saskatchewan.

**Fig. 2:**
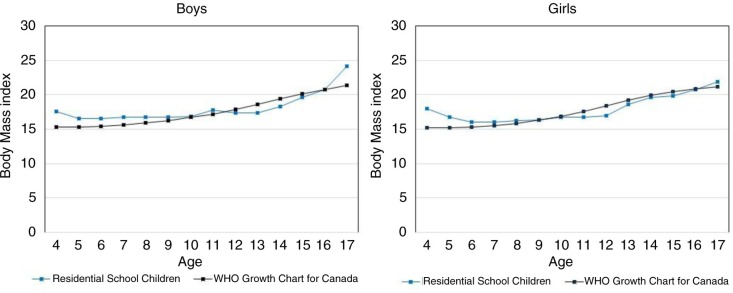
Body mass index – 50th percentile values by age and sex: children from residential schools and current WHO growth charts.

Figure 2 shows 50th percentile BMIs for boys and girls admitted to residential schools, and corresponding BMI values from current WHO growth charts for Canadian children, all by age. There was a trend for residential school children and youth to have slightly higher median BMIs at younger ages and slightly lower median BMIs at older ages but overall 50th percentile values were very similar to current age-specific BMI reference values.

**Fig. 3:**
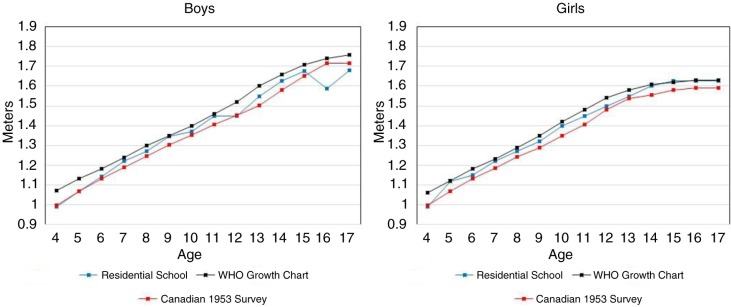
Height – 50th percentile values by age: children from residential schools, WHO growth charts, and 1953 Canadian survey.

Figure 3 shows the 50th percentile heights of residential school children and youth, and the corresponding values from the WHO growth charts and the Canadian 1953 height and weight survey. Residential school children and youth tended to be slightly taller than non-First Nations children from the 1953 survey, but slightly shorter than Canadian children today. Median weights of First Nations children (not shown) were similar to those from the 1953 survey.

## Discussion

On first entry to Manitoba and Saskatchewan residential schools from 1919 to 1953, First Nations children and youth from diverse Indigenous communities had substantially lower rates of overweight and obesity (10.9%) than both non-Indigenous (32%) and Indigenous (56.3%) children and youth today (1,9). Furthermore, they were more likely to have normal BMIs (80%) than their contemporary Canadian counterparts but higher rates of underweight, particularly those attending residential schools on the southern prairies. Importantly, WA Z-scores suggest that most of the study population exhibited good short-term health, while HA Z-scores also revealed satisfactory longer term health and nutrition (29). These findings not only provide a unique context of the current epidemics of obesity and type 2 diabetes among First Nations children and youth but also reveal new information reflecting the general nutritional condition of Indigenous children during the residential school era. Importantly, since most physical examinations were performed at the time of a child's first entry to residential school, our findings largely reflect conditions in the child's home community rather than the impact of the residential school system.

Only 2 publications report weight and/or height for Canadian children and youth prior to 1960 (16,17) but neither study included young First Nations people. The earliest published height and weight information for First Nations children and youth dates back to 1969 (30), but, as with the earlier studies, BMI, WA and HA cannot be calculated. More recently, there is detailed BMI information for Canadian children and youth (largely non-First Nations) that shows more than a doubling of overweight/obesity rates from the late 1970s to 2009–2011 (1,2). Recent BMI data for First Nations children and youth are more regional (3–8), but a recent meta-analysis reports a combined overweight/obesity prevalence in this demographic of 56.3% (9).

BMI data from the residential school era provide context to the current epidemics of obesity and type 2 diabetes among First Nations children and youth. First, with overweight/obesity affecting fewer than 11% of boys and girls on first entry to residential schools, these findings provide the earliest anthropometric evidence that the current obesity epidemic affecting this demographic is a recent phenomenon. Second, we speculate that our observations foreshadow what was to come. Not only was there a trend towards a gradual increase in overweight/obesity rates over successive time periods, but younger children aged 4–7 were most affected. This suggests a cohort effect in the progression of adiposity during a period of profound change in the way of life for First Nations people. These findings are also consistent with a recent study showing higher rates of BMI increase with more recent-born cohorts of First Nations people (31). Third, although differences between the sexes were small, girls in the older age groups already exhibited the higher overweight/obesity rates that have been observed more recently (5,6,8,9). Since both First Nations female adolescents and women have consistently manifested higher rates of type 2 diabetes than their male counterparts in recent decades (12,14), these residential school data suggest that the antecedents of these higher rates may have already begun to emerge at that time. Finally, First Nations girls also had the highest prevalence of underweight. If underweight persisted into child-bearing age, those affected had a higher risk of having low- birth weight infants. Although recent attention has focused on the macrosomia/diabetes link among First Nations peoples (32), low birth weight is also a predictor for type 2 diabetes (33), and continued to be a significant concern among First Nations people as recently as the 1970s (34).

Because our study is based on data gathered about First Nations children and youth on first entry to residential schools, it reflects the general nutritional conditions of children in their home communities rather than the nutritional impact of the residential school system. Nonetheless, as these unique data are from the residential school era, a few comments are warranted to provide perspective. The Truth and Reconciliation Commission (35), archival material (18,21,22) and recent reports of nutrition experiments in First Nations communities (36), all speak of the widespread hunger and malnutrition of First Nations children and youth attending residential schools. In contrast, this study provides evidence that, on first entry to residential schools on the prairies, most children and youth had normal BMI and WA reflecting good short-term health. Another finding is that these children and youth were more likely to be short rather than underweight for their age (i.e. 7.3% vs. 2.4% with >−2 Z-scores for HA and WA, respectively). But height differences may reflect the gradual overall increase in peoples’ height that has occurred in Canada over the past century. Indeed, children entering residential schools tended to be taller than their non-Indigenous counterparts in the 1953 Canadian survey, even though both groups had lower median heights than the contemporary WHO reference group (see Fig. 3). These data suggest good longer term health and nutrition for these First Nations children as well. Thus, insofar as these data reflect the nutritional conditions of First Nations communities in Manitoba and Saskatchewan, they suggest that reports of malnutrition among children and youth attending residential schools, on the prairies at least, can be largely attributed to living conditions within the residential schools themselves rather than to pre-existing malnutrition. One finding supporting that conjecture relates to older First Nations youth. Unlike younger children, they were more likely to have had previous residential school experience, and their higher rates of underweight could have resulted from a decline in caloric intake associated with residential school attendance.

Notwithstanding the above, overall rates of underweight are consistent with concerns about malnutrition in some First Nations communities during the study period. Although we were not able to compare the overall proportions of underweight First Nations children in this study with their non-First Nations contemporaries (e.g. it is conceivable that many non-First Nations prairie children may have been underweight as well, particularly during the Great Depression), it is troubling that almost 4% of all children and youth entering residential school were very underweight, that the highest rates of underweight occurred before and during the Great Depression, and that some schools (Brandon and Round Lake) had substantially higher rates of underweight than other schools. These findings strongly suggest that certain subgroups of children and youth and their communities were not receiving adequate foodstuffs and add objective support to archival reports and the personal recollections of First Nations people.

### Strengths and limitations

The strengths of this unique study include the large total number of children and youth for whom BMIs could be calculated, their age range, the eras covered, and the geographical and religious denominational mixture of residential schools. Although we do not know whether our data are nationally representative, the relative consistency of our findings between the sexes, among age groups, across a large geographical area, among children from diverse First Nations populations and from different residential schools suggest that the very high proportions of normal BMI and WA accurately reflect the condition of First Nations children on first leaving their home communities to attend residential schools in mid-western Canada. Its limitations primarily relate to the data source with its imprecision in age, the timing of physical examinations, the type (and likely consistency) of height and weight measurement devices and contextual information. Additionally, the numbers of very young and older children and youth were small, and we were unable to compare our BMI findings with non-First Nations children from the same periods. Moreover, the validation of BMI cut-points used in this study did not include data from Canadian Indigenous children. Importantly, it was not within the scope or capacity of this study to investigate the impact of other factors that might have had some impact on caloric intake over such a large geographical area and extended period of time. These include early European settlement of the plains (37), community-specific characteristics and contexts (such as local economy and food security), residential school factors (such as the exclusion of children with tuberculosis and possible changes in admission criteria over time) and national or global events (such as world wars, depression and pandemics). However, there is certainly potential to draw on these study data in further work with or by communities to provide more comprehensive accounts of specific contexts.

Finally, it is important to comment on the ethical questions posed by the analysis of data obtained from disenfranchised children. In this case, the medical examination of children on admission to residential schools was carried out to identify and exclude those who were unwell. It is not unusual for historical information in the public domain to be drawn into contemporary research studies. Regarding the residential school era, the information is typically from documentary or photographic material rather than from anthropometric data. Were we to request access to similar data for the purposes of research about First Nations children and youth today, we would be required to adhere to contemporary guidelines for research involving Aboriginal peoples (38). However, current guidelines are limited in their recommendations for the ethical use of historical data. Nonetheless, they are clear that it is important for the research context to uphold principles such as mutual respect and benefit, concern for welfare and justice. These principles are especially critical in guiding sensitivity towards the research use of data taken from individuals who may still be alive, and for their descendants. For these people, the legacy of the era and the information that emerges about it have personal, collective and political ramifications. We continue to grapple with this issue and would encourage further and more widespread discussion. In this study, we have attempted to be respectful and objective in our approach to conducting the research. To minimize any foreseeable harm to individuals and their communities, data were anonymized and aggregated. Finally, in the context of a literature on Indigenous peoples dominated by a discourse of poor health and outcomes, this study highlights evidence that the overwhelming majority of young First Nations children had normal anthropometric indices when they were taken from their home communities to attend residential schools.

## Conclusions

Most First Nations children and youth leaving their home communities to attend residential schools in Manitoba and Saskatchewan had normal BMI, WA and HA with lower rates of overweight/obesity and higher rates of underweight than their contemporary Canadian Indigenous and non-Indigenous counterparts. These historical findings provide insights and context to the current epidemics of obesity and type 2 diabetes among First Nations people and to the general nutritional status of children and their home communities during the residential school era.
